# Diagnostic Performance of a Novel Multiplex PCR Assay for Candidemia among ICU Patients

**DOI:** 10.3390/jof5030086

**Published:** 2019-09-17

**Authors:** Stefan Fuchs, Cornelia Lass-Flörl, Wilfried Posch

**Affiliations:** Institute of Hygiene and Medical Microbiology, Medical University of Innsbruck, 6020 Innsbruck, Austria

**Keywords:** molecular diagnostics, candidemia, yeast multiplex PCR, *Candida* PCR

## Abstract

Candidemia poses a major threat to ICU patients and is routinely diagnosed by blood culture, which is known for its low sensitivity and long turnaround times. We compared the performance of a novel, *Candida*-specific multiplex real-time PCR assay (Fungiplex^®^ Candida IVD Real-Time PCR Kit) with blood culture and another established diagnostic real-time PCR assay (LightCycler SeptiFast Test) with respect to *Candida* detection from whole blood samples. Clinical samples from 58 patients were analyzed by standard blood culture (BC) and simultaneously tested with the Fungiplex Candida PCR (FP) and the SeptiFast test (SF) for molecular detection of *Candida* spp. Compared to BC, the FP test showed high diagnostic power, with a sensitivity of 100% and a specificity of 94.1%. Overall diagnostic accuracy reached 94.6%. Using SF, we found a sensitivity of 60%, a specificity of 96.1%, and an overall diagnostic accuracy of 92.9%. The Fungiplex Candida PCR has shown good sensitivity and specificity on clinical samples of high-risk patients for direct detection of *Candida* species in whole blood samples. Together with conventional diagnostics (BC and antigen testing), this new multiplex PCR assay may contribute to a rapid and accurate diagnosis of candidiasis.

## 1. Introduction

Yeasts of the genus *Candida* represent the most prevalent group of fungal pathogens in humans. Predominantly in patients with impaired immune response or upon trauma, *Candida* species can turn from endogenous colonizers to invasive pathogens. This is of particular significance in hematological or transplant patients. The most prominent members of the family are *Candida* (*C.*) *albicans*, *C. glabrata*, *C. parapsilosis*, *C. tropicalis*, and *C. krusei,* accounting for >90% of invasive candidiasis or candidemia cases [1,2]. Some other important, yet infrequent, species are, for example, *C. guilliermondii*, *C. orthopsilosis*, *C. inconspicua, C. nivariensis*, and multi-resistant *C. auris* [3,4]. So far these species only constitute a small number of all invasive *Candida* infections [5], but these rare species might become increasingly important in the future, particularly with respect to resistance to antifungals [6]. Globally, the burden of invasive *Candida* infections, just like the burden of other invasive fungal infections, is rising [7,8,9]. For example, *Candida* is responsible for an estimated 2000–12,000 invasive fungal infections per year in Germany alone [10]. Apart from the high level of morbidity or mortality associated with these infections [11], this is also reflected by the high healthcare costs attributed to fungal disease [12]. The highest risk of nosocomial infections is observed for patients above the age of 65 with prolonged hospital stays [13]. Candidemia hence poses a major threat to patients in intensive care units (ICUs).

For a long time, blood culture (BC) has been the gold standard of blood stream infection diagnostics, despite its drawbacks of low sensitivity (approx. 50%) and long turnaround times (3–5 days) [14]. However, experts have questioned the reliability of blood culture as the gold standard with respect to fungal infection and the evaluation of clinical test parameters (sensitivity and specificity), particularly in comparison to non-culture based assays such as real-time PCR [15]. As a consequence, quick and reliable diagnosis will be the key to optimized therapy and reduced morbidity and mortality in Candidemia [16,17].

Over the last 10 years, a variety of non-culture-based methods have tried to overcome the limitations of conventional culture-based diagnostics. There are a number of commercially available and (more or less) established and recognized diagnostic tests for *Candida* detection in whole blood samples or serum. For instance, different antigen/antibody detection systems identifying *β*-D-glucan (BDG), *Candida* mannan, or *Candida albicans* germ tube antibody (CAGTA). In comparison, *Candida* nucleic acid detection by real-time PCR assays is mainly done using in-house protocols with varying sensitivities and specificities [18,19]. However, there are a few commercially available real-time PCR assays, such as the MycoReal *Candida* PCR test (Ingenetix) [20], the LightCycler SeptiFast test system (Roche Diagnostics, Basel, Switzerland), and the recently promoted Fungiplex Candida IVD PCR Kit (Bruker Daltonik, Bremen, Germany. The SeptiFast test (SF) is a highly multiplexed approach targeting the 25 most prevalent sepsis pathogens [21], which is routinely used across European microbiology laboratories. Apart from PCR methodology, alternative approaches for *Candida* detection are also available, such as the microarray technology of the CubeDx Sepsis test (CubeDx GmbH, St. Valentin, Austria). This test is based on amplification, hybridization, and detection of pathogen nucleic acids [22]. The T2-System (T2 Biosystems, Lexington, Massachusetts, United States) uses magnetic resonance analysis to confirm candidemia [23]. The advantages of such molecular approaches are the superior sensitivity and the speed compared to culture-based testing [15].

So far, none of the molecular assays have shown unambiguous results in terms of sensitivity and specificity, partly also due to the suboptimal comparator (i.e., BC). In particular, a direct detection of *Candida* spp. in the bloodstream of patients remains challenging because of low pathogen loads, high amounts of background DNA, and co-extracted substances perturbing or even inhibiting PCR reactions [24,25]. This fact is exemplified by the diagnostic parameters of the well-established SF assay, which varies broadly from 40% to 92% sensitivity and 54% to 96% specificity in the literature [21,26,27,28], depending on the patient cohort, underlying disease, and other inchoate factors. Data on the clinical performance of other PCR-based assays are very limited. For instance, the MycoReal *Candida* PCR, a research-use-only assay, is stated to have a very low level of detection (LoD) of 3 CFU per mL, but data on sensitivity or specificity are still lacking [29].

In this pilot study, we aim to assess the diagnostic performance of the Fungiplex Candida IVD PCR Kit in patients at risk of candidemia compared to conventional diagnosis (i.e., blood culture). Additionally, results were compared to SF, another multiplex PCR assay performed directly from whole blood samples.

## 2. Material and Methods

### 2.1. Study Design

**Figure 1:**
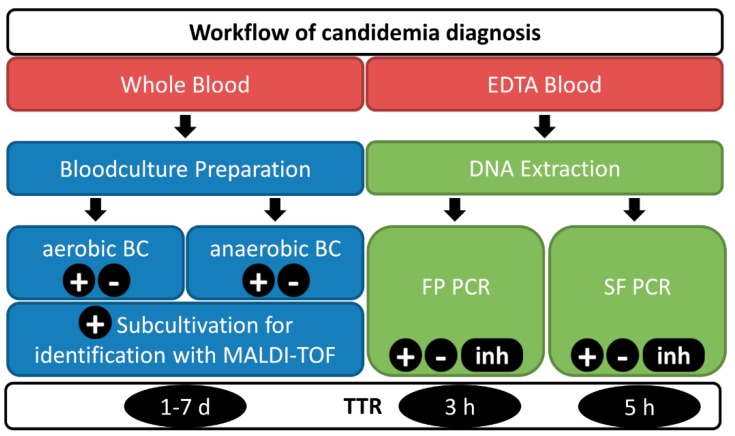
Diagnostic workflow including identification of Candida spp. in blood. Blood samples are sent for both blood culture and PCR-based diagnostics - Fungiplex (FP) and SeptiFast (SF). Ten milliliters of whole blood are injected into aerobic and anaerobic blood culture bottles. The SF test and FP PCR are run using the same DNA extract from 1 m*L* of EDTA blood. Positive blood cultures are subcultivated and analyzed using a MALDI-TOF system for species identification (ID). Results are reported as positive (+) including the identified species, negative (-) or, if PCR controls failed, as inhibited (inh). The time-to-result (TTR) varies between 1–7 days for BC (including ID) and 3–5 h for the molecular tests.

In this study, clinical samples (EDTA blood) from high-risk ICU patients suspicious of suffering from candidemia were collected between January 2018 and December 2018 and analyzed according to the workflow of candidemia diagnosis shown in Figure 1. Blood samples were directly sent to the Institute for Hygiene and Medical Microbiology at the Medical University of Innsbruck for candidemia diagnosis using both conventional BC- and PCR-based routine diagnostics (SF). Whole blood samples were taken by trained staff and used for the inoculation of the designated bottles for routine BC testing. At the same time, EDTA blood samples of these ICU patients were taken and processed for molecular detection using SF and Fungiplex Candida PCR (FP) PCR assays. The study was approved by the ethics committee of the Medical University of Innsbruck (Nr. 321/4.3, 8 April 2013). The workflow for BC- and PCR-based assays is shown in Figure 1. 

### 2.2. Patient Population

For this prospective, non-interventional evaluation of a novel multiplex PCR assay, the test results of 58 clinical samples from 54 patients were compiled and analyzed. The median age of the patients was 61.8 years (14.6–86.9) at the time of sampling. Out of these patients, 59% were male (*n* = 32) and 41% were female (*n* = 22). No additional demographic data or information on disease progression or outcome was recorded or analyzed. 

### 2.3. PCR-Based Assays

EDTA blood samples were collected from patients suspected of candidemia by trained staff in the ICUs of the University Hospital Innsbruck as recommended in the instructions of the LightCycler SeptiFast Test kit (Roche Diagnostics, Basel, Switzerland). From the collected EDTA blood, 1 mL was used to extract DNA using the MagNA-Lyser Instrument for mechanical lysis and the MagNA Pure Compact System (both Roche Diagnostics, Basel, Switzerland) with the corresponding Nucleic Acid Isolation Large Volume Kit I for DNA purification as specified in the manufacturer’s instructions. DNA was eluted in 200 µL and subsequently used for the molecular assays. The different panels of the two PCR tests are displayed in Table 1. While the FP detects *C. krusei* and *C. glabrata* specifically, the four additional species (C. *albicans, parapsilosis, tropicalis*, and *C. dubliniensis)* are summarized as *Candida* spp. The SF test can discriminate between five single *Candida* species (*C. krusei, C. glabrata, C. albicans, C. parapsilosis, C. tropicalis*). Single PCR test runs were performed for all samples.

The FP assay by Bruker was run according to the CE-IVD protocol of the kit. In brief, for each reaction, 10 µL of PCR Mastermix (MM) were supplemented with 1 µL of Candida MM and 3 µL of PCR-grade water before adding 1 µL of Internal Control Material and 5 µL of sample or control DNA. The final reaction volume of 20 µL was subject to PCR cycling on a Bio-Rad CFX96 real-time PCR device using CFX-Manager Software Version 3.1 (Bio-Rad Laboratories, Hercules, California, United States). Clinical samples were manually assessed for amplification signals in the four target channels (FAM for the internal control reaction and VIC, ROX, and Cy5 for *Candida* spp., *C. krusei*, and *C. glabrata*, respectively). Thresholds were manually adjusted according to the manufacturers´ instructions and result interpretation was done accordingly: internal controls (ICs) passed with Cq values > 20 (FAM channel) and samples were considered positive with Cq values < 45 (other channels). If no signal was detected and the corresponding IC failed, samples were reported as inhibited.

SF PCR was performed according to the manufacturers´ instructions. The PCR was performed on a LightCycler 2.0 device (Roche Diagnostics, Basel, Switzerland), LightCycler Software Version V4.1 (Roche Diagnostics). The interpretation of results was done using SeptiFast Interpretation Software (Roche Diagnostics, Version 2.0) and manually checked.

### 2.4. Blood Culture

A total of 20 mL of whole blood (10 mL for the aerobic and 10 mL for the anaerobic bottle) was drawn for one set of routine blood culture testing, as previously described [22]. BCs were incubated in the BACTEC FX system (Becton-Dickinson, Franklin Lakes, New Jersey, United States) for a maximum of 5 days. Positive BCs were further examined by gram-staining and microscopy. Sub-cultivation of retrieved pathogens on agar plates was done according to standard techniques [22]. Identification of the pathogens was performed by Matrix-Assisted Laser-Desorption Ionization–Time-Of-Flight (MALDI-TOF) using MALDI Biotyper^®^ system, MBT Compass Software IVD V4.2 (Bruker Daltonik, Bremen, Germany).

### 2.5. Statistical Procedures

Results of the molecular tests were compared against conventional BC and subsequent analysis in the MALDI Biotyper System (Bruker, Billerica, Massachusetts, United States) for identification of pathogens in positive blood cultures. This served as the gold standard of diagnosing blood stream infections. The results of the individual tests were compiled and concordant or discrepant results were used for the calculation of sensitivity, specificity, negative predictive value (NPV), positive predictive value (PPV), and overall diagnostic accuracy, as described elsewhere [30]. In the case of uninterpretable PCR results (e.g., due to inhibition of PCR amplification), the samples were excluded from the further analysis.

## 3. Results

A total of 58 EDTA blood samples from 54 ICU patients were assessed for the presence of *Candida* DNA and compared to BC. Five *Candida* infections (8.6%) were confirmed by BC within the selected patient group, while 53 cultures tested negative for *Candida* infection (91.4%, Table 2). 

The FP Kit identified eight samples as positive for *Candida* DNA (13.8%), comprising *C. glabrata* (*n* = 2) and *Candida* spp. (*n* = 6), and 48 samples as negative for *Candida* (82.8%). FP detected all culture-positive samples. Among the three positive FP samples that tested negative for *Candida* by BC, FP revealed *Candida* spp. (*n* = 2) and *C. glabrata* (*n* = 1). Overall, two samples displayed PCR inhibition (3.4%) and were therefore excluded from the subsequent statistical analysis. 

SF detected five samples (8.6%) that were positive for *Candida*, while FP identified eight *Candida*-positive samples. These three additional *Candida*-positive samples were detected as *Candida* spp. (*n* = 2) and *C. glabrata* (*n* = 1) by FP. The Cq-values for both PCR-based assays are shown in Table 2. For FP and SF, the Cq-values for BC-positive samples ranged from 32.59–38.38 and 34.19–39.11 and for BC negative samples from 30.69–33.19 and 33.64–33.82, respectively. SF detected *Candida* DNA in two of these culture-negative FP-positive samples. SF was negative in two culture-positive samples. For SF, we observed inhibited PCR reactions in five samples, whereas only two PCR amplifications were inhibited using FP. This represents a reduction of 60% of inhibited, and hence unresolved, samples.

In Table 2, the results of BC, the corresponding FP, PCR, and the SF test results are collated. Compared to SF, the FP kit showed slightly enhanced performance regarding inhibition in this study, as displayed in Table 2. Both non-culture-based assays concordantly yielded 48 negatives.

Based on the concordance between the PCR results and BC as the gold standard, sensitivity and specificity of the FP PCR were calculated as 100% and 94.1%, respectively. The positive predictive value (PPV) and the negative predictive value (NPV) of this novel multiplex PCR assay were 63% and 100%, respectively, resulting in an overall diagnostic accuracy of 94.6 %. A comparison of SF with BC results showed a sensitivity of 60%, a specificity of 96.1%, a PPV of 60%, a NPV of 96%, and an overall diagnostic accuracy of 92.9%.

## 4. Discussion

The diagnostic performance of the FP real-time PCR in this study was very promising, with 100% sensitivity, 94.1% specificity, and 94.6% diagnostic accuracy. Direct comparison of the diagnostic values with other published methods specific for detection of *Candida* is complex due to the unique settings of each clinical study. Nevertheless, one recent study assessing the T2Candida approach showed a lower specificity of <60% for high-risk ICU patients compared to data shown here [23]. Another study reported a sensitivity of 43% and a specificity of 94% for the T2 system for a patient cohort receiving empirical antifungal treatment [31]. However, our study suffers from a limited sample size. 

For the well-accepted mannan antigen test, sensitivities of 58% and specificities of 93% are reported [32]. The performance characteristics of the SF multiplex PCR (60% sensitivity, 96% specificity) were consistent with published results [22,28]. 

Although BC is considered the gold-standard for *Candida* diagnostics of bloodstream infections, and as such was used as the index test for this study, its sensitivity is known to be low (~50%). PCR-based assays typically show superior sensitivities in the detection of *Candida* spp. compared to BC. The discrepancy between BC- and PCR-based assays in the detection of *Candida* spp. was not due to antifungal treatment of patients, since these patients did receive antibiotics, but no antifungals. We detected higher Cq-values for both PCR-based assays in BC-negative samples (Table 2), which could explain a lower detection of *Candida* ssp. by BC. Empirical therapy of patients with prolonged hospitalization and an increased risk of candidemia in ICUs contributes to the fact that BC sensitivity drops markedly upon antifungal medication [33]. Prophylactic treatment with antifungals may hamper or prevent growth in blood culture but may not negatively influence the performance of the molecular tests [18]. Instead, antibiosis could actually foster PCR-based detection via endogenous destruction of the fungal cells in the body and following release of fungal DNA. A similar mechanism has been postulated, for example, for *Aspergillus fumigatus* and the detection of its DNA in blood or serum [34]. Clinical criteria were not included in this study, although both molecular tests were positive in two out of three culture-negative cases that yielded a positive PCR result. The lower limit of detection (LoD) is stated by the manufacturers to be 30 genome equivalents or CFU/mL for both the FP and SF PCRs, respectively. Hence, the molecular detection is equally sensitive.

Inhibition of PCR reactions is a critical aspect of molecular test reliability, potentially generating false negative results. It is known that there are a number of substances (e.g., heparin) that are routinely used in ICU wards and which concurrently have been shown to negatively affect or even disrupt PCR kinetics and hence a successful detection of pathogens [35]. This is particularly true for blood or blood-derived samples [36]. Consequently, any improvements in terms of PCR stability are highly desirable. In this study, the SF test was inhibited in 8.6% (*n* = 5) of the samples tested. This is consistent with data reported in other studies [37]. Using the FP PCR, the number of inhibited PCR reactions was clearly reduced by 60%. Thus, PCR inhibition might be a minor problem for the FP assay compared to other multiplex PCR tests used in routine laboratory settings.

One crucial aspect of molecular pathogen detection is the extraction of germ DNA from sample material. Since there are multiple options for manual DNA extraction as well as various automated platforms available, the compatibility of individual tests with a selected extraction method is very important and directly influences the diagnostic parameters (sensitivity and specificity) [38]. FP was validated by two different extraction systems, which were not available for the current study. The manufacturers have also used smaller volumes (400/500 µL) of whole blood or serum for DNA extraction and analysis. These differences within the DNA extraction process can impact test results of individual labs and hinder inter-lab reproducibility.

Since it can be of clinical importance to distinguish individual *Candida* species [39], the read-out of the FP assay summarizing four quite common *Candida* species as *Candida* spp. is suboptimal. Hence, also in the light of changing antifungal resistance patterns [40], a more specific detection of single species might be desirable. Interestingly, for one sample the FP assay was positive (*Candida* spp.) while culture showed growth of *C. guilliermondii*. As this species is not listed in the panel of FP, it is not clear whether this is evidence for a co-infection with *C. guilliermondii* and another *Candida* species detected by the PCR, or if this is due to a serendipitous detection of the PCR for this species.

In conclusion, this pilot study using a multiplex PCR assay for the detection of candidemia among high-risk ICU patients showed a high sensitivity, specificity, as well as overall diagnostic accuracy. Although the PCR methodology used in the current study provides the first evidence of a diagnostic advantage in this patient group compared to BC, a multicenter study with higher patient and sample numbers would be necessary to substantiate these findings. Nonetheless, multiplex PCR assays such as Fungiplex Candida can help in optimizing treatment algorithms based on the prompt and reliable information for the clinicians.

## Figures and Tables

**Table 1:** Candida species covered by the molecular tests used in this study.

FP PCR		SF PCR
*Candida krusei*		*Candida krusei*
*Candida glabrata*		*Candida glabrata*
*Candida* spp. incl.	*C. albicans*	*Candida albicans*
	*C. parapsilosis*	*Candida parapsilosis*
	*C. tropicalis*	*Candida tropicalis*
	*C. dubliniensis*	

In the FP assay, *Candida krusei* and *C. glabrata* are detected in individual channels; *C. albicans, C. parapsilosis, C. tropicalis* and *C. dubliniensis* are detected in one channel and are reported as *Candida spp*. In the SF test the five species listed are identified individually, while *C. dubliniensis* is not detected at all.

**Table 2:** Summary of test results comparing blood culture (BC) to the molecular detection by Fungiplex (FP) and SeptiFast (SF) PCR tests.

Result (*Candida*)	BC	FP		SF	
**POS**	5	8	Cq	5	Cq
	*Candida albicans*	*Candida* spp.	32.59	*Candida albicans*	30.69
	*Candida albicans*	*Candida* spp.	33.42	*Candida albicans*	31.04
	*Candida glabrata*	*Candida glabrata*	35.31	*Candida glabrata*	33.19
	*Candida dubliniense*	*Candida* spp.	36.30	neg	n/a
	*Candida guillermondii*	*Candida* spp.	38.38	neg	n/a
	neg	*Candida* spp.	37.17	*Candida albicans*	33.82
	neg	*Candida* spp.	39.11	*Candida albicans*	33.64
	neg	*Candida glabrata*	34.19	neg	n/a
**NEG**	53	48		48	
**inh (PCR)**	*n*/a	2		5	
**total**	*n* = 58				

Samples positive (POS) or negative (NEG) for *Candida* are listed correspondingly for each individual test. Cq-values (Cq) of Candida-positive samples are provided for the FP and SF assays. Inhibited (inh) PCR reactions are also indicated. n/a, not applicable.
